# Preconditioning with Wound Fluid Enhances Immunosuppressive Properties of Mesenchymal Stromal Cells In Vitro

**DOI:** 10.3390/ijms26010293

**Published:** 2024-12-31

**Authors:** Helena Moratin, Isabel Mache, Miguel Goncalves, Totta Ehret Kasemo, Manuel Stöth, Till Jasper Meyer, Stephan Hackenberg, Agmal Scherzad, Marietta Herrmann

**Affiliations:** 1 Department of Oto-Rhino-Laryngology, Head and Neck Surgery, University Hospital Würzburg, Josef-Schneider-Str. 11, D-97080 Wuerzburg, Germany; 2 IZKF Research Group Tissue Regeneration in Musculoskeletal Diseases, University Hospital Wuerzburg, D-97070 Wuerzburg, Germany; 3 Department of Orthopedics, Bernhard-Heine-Center for Locomotion Research, University of Wuerzburg, D-97074 Wuerzburg, Germany

**Keywords:** MSC, PBMC, wound fluid, immunomodulation, wound healing

## Abstract

Immunosuppression is one key feature of mesenchymal stromal cells (MSCs) that has high expectations for therapeutic use. The influence of pro-inflammatory stimuli can modify the characteristics of MSCs and enhance immunosuppressive properties. The local postoperative environment contains cytokines, MSCs, and immune cells in high quantities, and their mutual influence is still unclear. Knowledge of in vivo processes is pivotal for potential therapeutic applications, and therefore, the aim of this study was to investigate the influence of wound fluid (WF) on the immunomodulatory potential of MSCs. CD4+ cells were co-cultured with native or WF-preconditioned MSCs for 5 days. CFSE staining revealed significant suppression of T cell proliferation after co-culture that was even more distinct in co-culture with WF-MSCs. The concentration of IDO-1, TGF-β1 and IFN-γ was higher while TNF-α was reduced in co-culture supernatants, indicating a transition to an anti-inflammatory milieu. In summary, the results provide evidence that the influence of WF alters the immunomodulatory potential of MSCs. These findings should serve as the basis for further investigations with a focus on T cell subpopulations.

## 1. Introduction

There is exceptional scientific interest in mesenchymal stromal cells (MSCs) as they bear promising potential in a broad spectrum of research areas. In general, MSCs are considered to be multipotent cells with self-renewing differentiation capacity and a fibroblast-like morphology that can differentiate into various mesenchymal lineages of tissue cells, such as adipocytes, chondrocytes or osteoblasts [1]. Several tissue sources have been described from which MSCs can be isolated, including adipose tissue, umbilical cord, dental pulp, and bone marrow [2,3]. The capability to mediate immunosuppression is a distinct feature of MSCs. In the context of hematopoietic cell transplantation, for example, dysfunctional immune response can elicit activated T cells attempting to eradicate antigen-bearing cells of the host. This condition is referred to as graft-versus-host disease (GvHD) and can result in severe organ damage. The use of MSCs for the treatment of GvHD has already found its way into clinical procedures, and study data indicate that MSCs can reduce the incidence and severity of GvHD [4,5]. The immunomodulatory properties of MSCs make an application to treat various diseases seem possible, from inflammatory and autoimmune diseases such as multiple sclerosis to metabolic conditions such as diabetes [6] and even cancer therapy [7].

The immunosuppressive activity of MSCs is, on the one hand, mediated by direct cell-to-cell contact with different subsets of immune cells such as T and B cells, natural killer (NK) cells, neutrophils, macrophages, monocytes, and dendritic cells (DC) [8]. In particular, suppression of B and T lymphocyte proliferation and stimulation of regulatory T cells (Treg) differentiation under the influence of MSCs have been described. In addition, the activity of macrophages and neutrophils as components of innate immunity can be suppressed in their activity [9,10]. On the other hand, the secretion of cytokines, growth factors, and chemokines, collectively referred to as the secretome, plays an important role in the mechanisms of MSC-associated immunomodulation. Transforming growth factor-β1 (TGF-β1), tumor necrosis factor-α (TNF-α), interferon-γ (IFN-γ), and indoleamine 2,3-dioxygenase (IDO) are among the main drivers of this process [11]. These paracrine factors may be encapsulated in so-called extracellular vesicles (EVs), which are secreted by the cells. EVs contain many bioactive molecules, including lipids, surface receptors, enzymes or RNA particles, and act as a communication medium and even drug delivery vehicles between cells [12]. They have the potential to be cell-free therapeutic agents, as the cell-associated immunogenic side effects can be minimized [13]. The immunoregulatory influence of EVs is similar to that of the MSCs from which they are derived. However, the paracrine effect may vary depending on the source of the MSCs, the target cells, and the microenvironment surrounding the cells [14].

The process of wound healing is a complex interplay between cellular and non-cellular components. It involves highly dynamic, interdependent mechanisms characterized by hemostasis, inflammation, proliferation, and tissue remodeling. Immediately after the onset of trauma, numerous cytokines and chemokines are secreted to attract various cell types to the wound site, including immune cells and MSCs [15]. MSCs contribute to all stages of the physiological wound-healing process by regulating the local immunological milieu, promoting angiogenesis, and reducing inflammation [16]. Studies have shown enhanced tissue regeneration through the application of MSCs, especially in cases of chronic wounds with poor circulation. These beneficial effects have already been translated from the in vitro situation and confirmed in preclinical and clinical studies [17,18,19,20,21]. However, the therapeutic potential of MSCs is often restricted due to limited cell survival and low engraftment potential, which requires an improvement in efficacy [22]. The secretome of MSCs has pro-regenerative effects caused by inhibition of cell death and fibrosis [23], stimulation of vascularization [24], promotion of tissue remodeling, immunomodulation, and recruitment of other cells [25]. The conditions under which MSCs are cultured are a critical factor in the composition of their secretome. Preconditioning with hypoxia and immunomodulatory factors is, therefore, a promising approach to improve therapeutic efficacy [26].

Most studies have investigated the influence of distinct stimulatory factors such as IFN-γ or TGF-β1 on MSC with the aim of defining specific parameters of preconditioning to improve therapeutic application [27,28,29]. However, it is also essential to understand how intrinsic stimuli can influence the function of MSCs. Previous studies have shown that microenvironmental signals associated with bone healing or platelet-derived factors can modulate the functional properties of MSCs [30,31]. In the local postoperative environment, various pro-inflammatory factors, as well as MSCs and cells of the immune system, accumulate in high amounts. The composition of wound fluid (WF) has been previously investigated [32,33]. This setting seems particularly relevant in the context of oncologic diseases, where the question arises whether surgical intervention can induce local immunosuppression. This could hypothetically lead to reduced anti-tumor activity against residual tumor cells, resulting in early recurrent tumor growth. Therefore, the aim of this study was to investigate whether preconditioning of MSCs with WF influences their immunomodulatory potential.

## 2. Results

### 2.1. MSC Characterization

**Figure 1:**
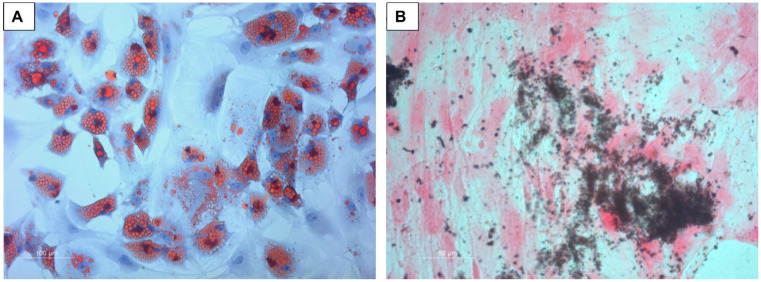
Microscopic analysis of the adipogenic and osteogenic differentiation capability of MSCs. (**A**) shows intracellular lipid droplets after Oil Red O staining, and (**B**) shows calcium mineral formation by von Kossa staining.

**Figure 2:**
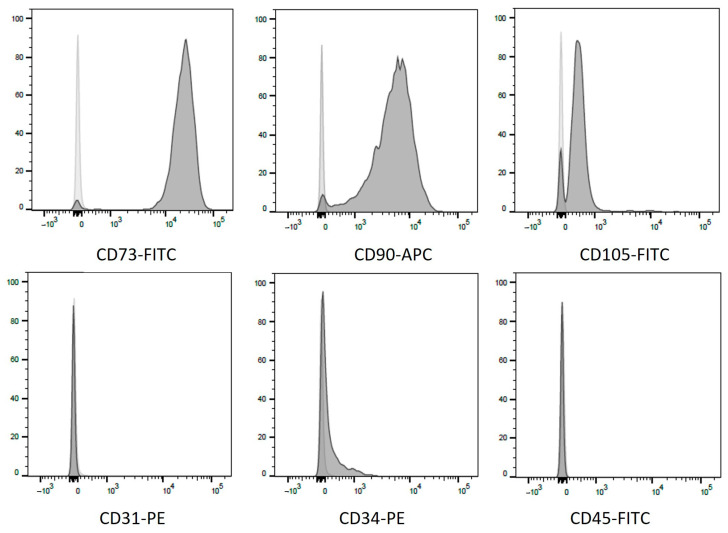
Flow cytometric analysis of MSC-specific surface markers. Positive expression of CD73, CD90 and CD105 is demonstrated. The lower row shows the analysis with proof of the absence of CD31, CD34 and CD45. Light grey curves depict corresponding isotype controls.

The differentiation capability of MSCs was confirmed by Oil Red O and von Kossa staining, as shown in Figure 1. Flow cytometry revealed the presence of CD73, CD90, and CD105 and the absence of CD31, CD34 and CD45 (Figure 2).

### 2.2. Suppression of Cell Proliferation

**Figure 3:**
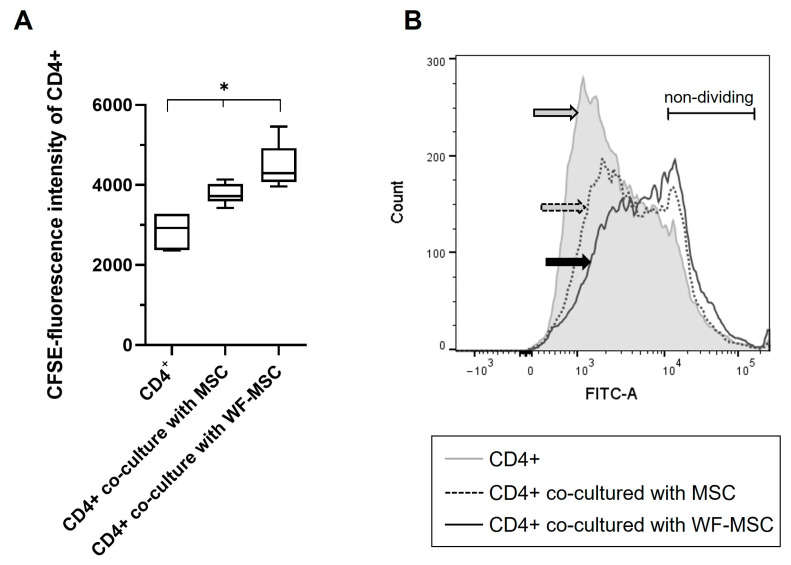
(**A**) Presentation of the CFSE proliferation measurements of CD4+ cells after co-culture with native MSCs or WF-preconditioned MSCs. As the data was normally distributed, an ANOVA with a Bonferroni post hoc test was performed. Data show the Median of the CFSE fluorescence intensity, with a lower fluorescence signal implying a higher proliferation rate. n = 6 individual experiments from different WF donors. As the measured CFSE fluorescence intensity signal was much higher in two specimens, data from these two were normalized. Asterisks indicate statistically significant results. Data are presented with box plots, the margins of which illustrate the 25th and 75th percentiles. (**B**) Exemplary histogram of CFSE-stained CD4+ T cells of one experiment. Grey line = CD4+ T cells as a control group; dashed line = CD4+ T cells co-cultured with MSCs; solid line = CD4+ T cells co-cultured with WF-stimulated MSCs. Arrows mark the different subpopulations. FITC (x-axis) shows the fluorescence intensity of CFSE-stained cells. Y-axis = number of events measured. * *p*-values ≤ 0.05.

CFSE staining was performed to determine the proliferation of CD4+ cells. A lower fluorescence signal indicates higher rates of cell proliferation. The proliferation of CD4+ cells upon PHL stimulation was significantly suppressed after co-cultivation with MSCs. Moreover, the proliferation rate differed significantly between the native MSCs and the WF-preconditioned MSC group. Results are shown in Figure 3. Figure 3A shows the median fluorescence intensity of 6 WF donors, and Figure 3B represents an exemplary histogram for the CFSE fluorescence signal with overlays of the different experimental groups. It is shown that the proliferation rate is the lowest in CD4+ cells that were co-cultured with WF-stimulated MSCs (solid line, black arrow). 

### 2.3. Cytokine Gene Expression in MSCs After WF Incubation

**Figure 4:**
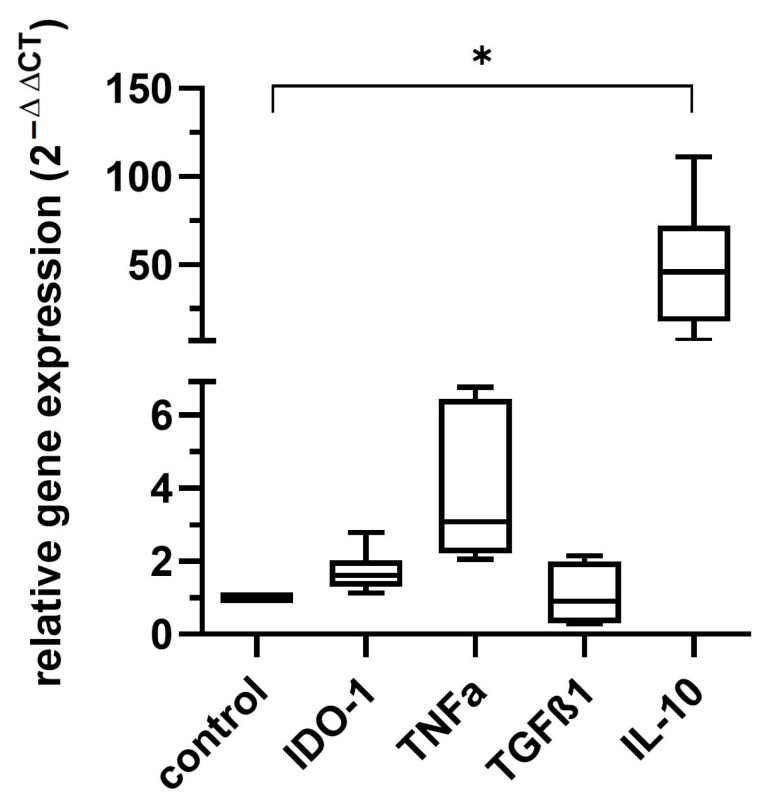
Results of the quantitative Real-Time PCR of IDO-1, TNF-α, TGF-β1, and IL-10 in MSCs after incubation with 30% WF for 3 h. The housekeeping gene/control was GAPDH. As the data was normally distributed, an ANOVA with a Bonferroni post hoc test was performed. Gene expression increased for the markers TNF-α and IL-10. Only the alteration of IL-10 was statistically significant, as indicated by an asterisk. Data are presented with box plots, margins of which illustrate the 25th and 75th percentiles n = 6 individual experiments from different WF donors, each measurement performed as triplicate. * *p*-values ≤ 0.05.

The mRNA levels of TNF-α, TGF-β1, IL-10 and IDO-1 were measured in MSCs after cultivation with 30% WF for 3 h by quantitative Real-Time PCR. As data are presented as 2^−ΔΔCT^, MSCs cultured with standard medium (control) were set to 1. Figure 4 shows that IL-10 and TNF-α expression increased compared to normal culture conditions. Mean values with standard deviation were 1.71 +/− 0.57 for IDO-1, 3.93 +/− 2.07 for TNF-α, 1.08 +/− 0.79 for TGF-β1 and 48.55 +/− 36.32 for IL-10. Results were only statistically significant for IL-10 in comparison to the control.

### 2.4. Cytokine Quantification in Co-Culture Supernatants

**Figure 5:**
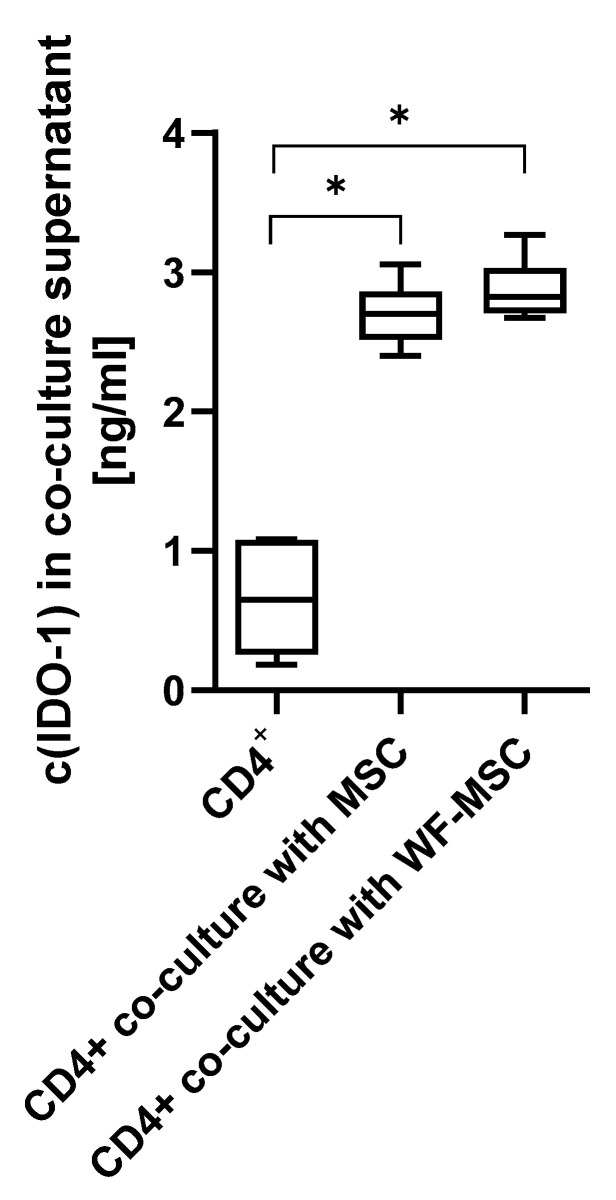
The concentration of IDO-1 in supernatants after 5 days in culture. CD4+ cells alone or in co-culture with MSCs or WF-preconditioned MSCs. n = 6 individual experiments from different WF donors, each measurement performed as duplicate. As the data was normally distributed, an ANOVA with a Bonferroni post hoc test was performed. Asterisks indicate statistically significant results. * *p*-values ≤ 0.05.

To measure the amount of cytokines, an ELISA for IDO-1 and a multi-analyte flow assay for TNF-α, TGF-β1, IFN-γ, and IL-10 were performed. As shown in Figure 5, the concentration of IDO-1 was significantly higher in the co-culture groups compared to CD4+ cells alone. There was no significant difference between the MSC subgroups.

**Figure 6:**
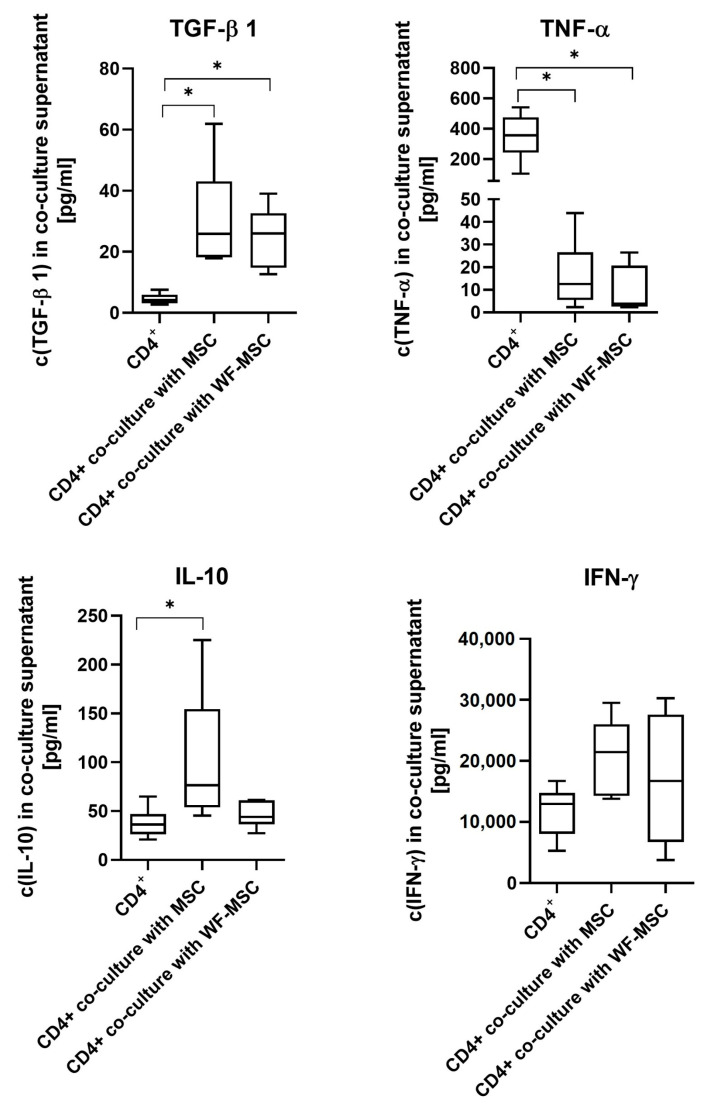
Results of multi-analyte flow assay for the cytokines TNF-α, TGF-β1, IFN-γ, and IL-10 in supernatants of CD4+ cells alone or after 5 days in co-culture with native MSCs or WF-preconditioned MSCs. n = 6 individual experiments from different WF donors, each measurement performed as duplicate. Data were normally distributed for TGF-β1, IFN-γ, and IL-10, and an ANOVA with a Bonferroni post hoc test was performed. In the case of TNF-α, values were not normally distributed, and the Kruskal–Wallis test with Dunn’s post hoc was performed. Asterisks indicate statistically significant results. Data are presented with box plots, the margins of which illustrate the 25th and 75th percentiles.

Cytokine expression varied between the analyzed cytokines, as shown in Figure 6. There were significant differences between native MSC co-culture and CD4+ single cultures for all cytokines except IFN-γ. Concentration was higher for IL-10 and TGF-β1, whereas TNF-α was significantly less. For TGF-β1 and TNF-α, the concentration between CD4+ single cultures and WF-MSCs differed significantly. There was a tendency for co-culture supernatants with native MSCs to contain more IL-10 than with WF-MSCs. However, there was no statistical significance. 

## 3. Discussion

The implementation of MSC-based therapies is the subject of high expectations in many areas of medical care. Krampera et al. outlined three main principles that characterize the beneficial effects of MSCs in vivo: cell expansion and differentiation into different mesodermal tissue types, modulation of the immunological microenvironment through intercellular interaction via cell-to-cell contact and paracrine signaling, and modification of phagocyte polarization and micro milieu composition by apoptosis involving MSCs and immune cells [34]. Specific preconditioning of MSCs aims to improve these properties for a targeted, safe, and reproducible therapeutic application. The secretome and, consequently, the immunomodulatory potential of MSCs are highly dependent on environmental influences, and most studies have focused on the effect of defined stimuli such as prostaglandin E2 [35], TGF-β1 [36], TNF-α or IFN-γ [37]. However, a comprehensive knowledge of the influence of physiological conditions on MSC properties, e.g., in the context of wound healing, is essential, especially in consideration of possible therapeutic applications. The aim of this study was to investigate how physiological WF affects the interaction between MSCs and T cells, a crucial interaction in the initial postoperative period. To better understand the underlying processes, the supernatants of the media were analyzed for cytokine composition.

WF from the local postoperative environment contains high levels of cytokines, but the composition varies depending on the time of wound healing. The WF used in this study was collected from drains, which were usually removed on the second day after surgery. Our group’s previous cytokine composition was WF [32]. Major components include IL-6, IL-8, monocyte chemoattractant protein-1 (MCP-1/CCL2), RANTES (CCL5), angiogenin, insulin-like growth factor binding protein-2 (IGFBP-2), neutrophil-activating peptide-2 (NAP-2), osteopontin and tissue inhibitor of metalloproteinases-2 (TIMP-2). MSCs have been shown to suppress the proliferation of CD4+ and CD8+ T cell subsets with a shift from pro-inflammatory Th1 to anti-inflammatory Th2 cells [38]. Furthermore, the generation of Tregs is induced [39]. This immunological transformation is accompanied by a change in the inflammatory cytokine milieu [40]. In this study, CD4+ T cells were used as responders to avoid the potential influence of the non-T cell components of PBMCs. However, in this setting, interactions between T cell subpopulations are excluded, which could have a relevant influence on the results and should be considered in further studies. Moreover, the functionality and activation state of immune cells could be examined by measuring markers like CD25 or CD44. The data in the present study confirmed a transition to an anti-inflammatory milieu by co-culture of T cells and MSCs, resulting in higher expression of IDO-1, TGF-β1, IL-10 and IFN-γ and suppression of TNF-α. This led to a reduction in the proliferation rate of CD4+ cells. This effect was even more pronounced after MSC preconditioning with WF. MSC-induced Treg generation is a consequence of transformation from conventional T cells [41]. The process appears to depend on the presence of TGF-β1 and monocyte interaction [39]. Analyzing the distribution of T cell subpopulations after incubation with WF could be an interesting focus for further investigations.

One of the main drivers of MSC-associated immunosuppression is IDO. The enzyme degrades tryptophan, which leads to an attenuation of T cell proliferation due to metabolic starvation [42]. Previous studies have shown that IFN-γ enhances the immunosuppressive effect of IDO [43]. In this study, IDO-1 was highly expressed after the co-culture of MSCs with T cells. However, WF did not significantly alter the gene expression of IDO-1 in MSCs. The amount of IDO-1 measured in the supernatants after co-culture did not differ between native MSCs and MSCs preconditioned with WF. This suggests that IDO-1 expression is dependent on T cell-associated stimuli and that WF has a limited effect on its expression in MSCs. As an additional investigation, the activity of inducible Nitric Oxide Synthase in MSCs could be evaluated, as it represents another key enzyme in this context. TNF-α is mainly produced by immune cells and is considered to be one of the most important factors in the microenvironment of inflammatory diseases. Deng and colleagues reported that MSCs inhibited the expression of TNF-α in T lymphocytes [43]. Accordingly, in this study, TNF-α was significantly reduced in supernatants after co-culture, although gene expression was elevated in MSCs after incubation with WF. There was no difference in measured TNF-α between unstimulated and WF-stimulated MSCs. IL-10 acts as an anti-inflammatory component by inhibiting the release of pro-inflammatory cytokines, including IFN-γ, IL-2, and TNF-α [44]. Interestingly, IL-10 mRNA expression was highly stimulated in MSCs 3 h after incubation with WF. However, there was a tendency for the concentration of IL-10 to be lower in the supernatants of co-culture with WF-preconditioned MSCs compared to native MSCs. This could be explained by the short half-life of IL-10. Furthermore, the quantitative real-time PCR measurements only provide information on the mRNA level at a given time point (3 h) and might thus not necessarily correlate with the protein levels detected in supernatants. In summary, there was no significant difference in measured concentrations of the cytokines in supernatants between native and WF-preconditioned MSCs. For this study, cytokines that seemed relevant against the background of wound healing were selectively investigated. In order to identify crucial factors that cause the stronger suppression of T cell proliferation by WF-preconditioned MSCs, the number of cytokines examined could be expanded.

One treatment modality for head and neck squamous cell carcinoma (HNSCC) is based on surgical resection followed by adjuvant radio(chemo)therapy with guidelines recommending the initiation of adjuvant therapy within 4–8 weeks. However, despite multimodal therapy, approximately 30% of patients ultimately experience locoregional failure. Moreover, there is a subset of tumors that have a relatively short interval until structurally detectable recurrence, which is associated with poor outcomes. Rapid recurrence is defined as significant tumor growth after oncologically adequate surgical resection but before the initiation of timely adjuvant therapy. This phenomenon is still not fully understood and is thought to involve tumor biological factors in addition to the quality of surgery [45]. There is increasing evidence that IL-10 can induce anti-tumor effects in an immune-dependent manner [46]. It has been shown that sustained elevated serum concentrations of IL-10, as achieved by PEGylation, can induce enhanced cytotoxicity and expansion of tumor-specific CD8+ T cells in animal studies [47]. The data from this study suggest that WF exposure has an inhibitory effect on the secretion of IL-10 in MSC-T lymphocyte co-culture. Hypothetically, in the setting of the local environment after oncologic surgery, this could lead to reduced immunologic anti-tumor activity, which may promote the occurrence of early local recurrences. To further elucidate this question, a setting involving cytotoxic T lymphocytes would be particularly interesting, and the next step would be the translation to an interaction with malignant cells.

Immunomodulation is a known property of MSCs with therapeutic potential. Specific modifications are necessary to facilitate a targeted application, and preconditioning of the cells represents a possibility of influencing the characteristics of MSCs. Preconditioning describes the treatment of cells that are carried out during the in vitro expansion of MSCs as part of cell therapies. However, this study was designed to mimic the conditions of the local postoperative environment, focusing on the immunological milieu and T cell proliferation. Therefore, WF from drains after oncologic surgeries was used to precondition MSCs. The results showed an attenuation of CD4+ cell proliferation after co-culture with MSCs, which was further enhanced by preconditioning the MSCs with WF. Co-culture supernatants of native MSCs contained significantly more TGF-β1 and IFN-γ and less TNF-α compared to CD4+ alone.

## 4. Materials and Methods

### 4.1. Isolation and Cultivation of Cells

#### 4.1.1. Human Mesenchymal Bone Marrow Stem Cells (BMSC)

The procedure of cell isolation from residual acetabular reaming material from patients receiving total hip arthroplasty has been described previously [48]. The Ethics Committee of the Medical Faculty of the University of Wuerzburg approved the study (91/19-me), and patients gave their informed consent. Isolated cells were further cultured in 75 cm^2^ tissue culture flasks (Greiner Bio-One GmbH, Frickenhausen, Germany) in a 15 mL cell culture medium to a confluence of approximately 90%. The stock expansion medium was composed of Dulbecco’s Modified Eagle Medium (DMEM, Life Technologies Corp. (Gibco), Carlsbad, CA, USA), 1% penicillin/streptomycin (Sigma Aldrich, Steinheim, Germany), and 10% fetal calf serum (FCS) (Linaris, Wertheim, Germany). Incubation was carried out at 37 °C and 5% CO_2_ overnight. This was followed by a medium change in DMEM + 10% Elarem (PL BioScience GmbH, Aachen, Germany) with subsequent further incubation in the incubator. The DMEM/Elarem medium was changed twice weekly. Traditionally, MSCs were defined by the expression of classical surface markers such as CD73, CD105, and CD90, along with the absence of markers like CD34, CD45, CD14 or CD11b, CD79alpha or CD19, and HLA-DR. However, more recently, there has been growing consensus that additional markers are necessary to define MSCs functionally, considering their diverse origins and source-specific characteristics [49]. In this study, cells of three different donors in passage 3/4/5 were used for the experiments.

#### 4.1.2. Characterization of MSC

To confirm the criteria for the definition of MSCs, tests for multidifferentiation capacity and classical cell surface marker expression were conducted. Osteogenic differentiation was performed by culturing 1 × 10^4^ cells/well in a 24-well plate (BD Falcon, Heidelberg, Germany) until a confluence of 70% was reached. The expansion medium DMEM-EM additionally contained 100 nM dexamethasone, 10 mM β-glycerophosphate and 0.2 mM ascorbate-2-phosphate (all Sigma-Aldrich). The von Kossa method was used to show the presence of calcium mineral components. Adipogenic differentiation medium consisted of DMEM-EM with 1 µM dexamethasone, 500 µM 1-Methyl-3-Isobutylxanthin, 100 µM Indomethacin and 1 µg/mL recombinant human insulin (all Sigma-Aldrich). Intracellular lipid droplets were stained with Oil Red O.

Flow cytometry was used to confirm surface antigen markers. MSCs were incubated with anti-CD31, anti-CD34, anti-CD45, anti-CD73, anti-CD105, and anti-CD90 (all antibodies were purchased from BD Bioscience, Heidelberg, Germany). Cell surface analysis was performed by flow cytometry (FACSCanto™; BD Bioscience, Heidelberg, Germany).

#### 4.1.3. Peripheral Blood Mononuclear Cells (PBMCs)

PBMCs were isolated from leukoreduction system chambers (LRSC), which were obtained from the Institute for Transfusion Medicine and Haemotherapy of the University Hospital Würzburg. LRSC contains concentrated leukocytes and is collected as a by-product of the manufacture of platelet concentrate by platelet apheresis procedures. The contents of the LRSC were transferred to a 50 mL centrifuge tube (Greiner Bio-One GmbH), which was filled up to 20 mL with 1× phosphate-buffered saline (PBS, Roche Diagnostics, Mannheim, Germany). A 15 mL lymphocyte separation medium (Lymphocyte Separation Media, Anprotec, Bruckberg, Germany) was used to separate the components, with the PBMCs located within a white ring below the blood plasma after 30 min centrifugation at 800× *g*. This layer was collected using a glass Pasteur pipette, transferred to a new 50 mL tube, and 1× PBS was added to a total volume of 50 mL. The cell suspension was centrifuged at room temperature for 10 min at 1400 rpm/394× *g,* and the supernatant was removed. Erythrocytes were eliminated by adding 5 mL erythrocyte lysis buffer (RBC erythrocyte lysis buffer, Thermo Fisher Scientific, Carlsbad, CA, USA). The tube was slowly shaken by hand for 3 min before a centrifugation period at 1400 rpm for 10 min. A total of 30 mL PBS was added, and centrifugation continued for another 10 min. The supernatant was removed, and cells were resuspended in 50 mL PBS, counted and used for further experiments. In total, PBMCs of three different donors were used.

#### 4.1.4. Enrichment of CD4+ Lymphocytes

To enrich the CD4+ cells from the entity of PBMCs, CD4 Microbeads (Miltenyi Biotec, B.V. and Co. KG, Bergisch Gladbach, Germany) were used according to the manufacturer’s protocol. The cell suspension with bound microbeads was placed on a MS Column (Miltenyi Biotec) and set in the magnetic field of the MiniMACS Separator (Miltenyi Biotec). CD4 and viability staining (APC anti-human CD4, BioLegend, San Diego, CA, USA; FVD 780 Fixable Viability Dye eFluor, Life Technologies Corp., Carlsbad, CA, USA) was performed according to the manufacturer’s protocols and analyzed by flow cytometry (BD FACSCanto^TM^ flow cytometer, Heidelberg, Germany) to check for cell vitality and efficiency of cell enrichment.

### 4.2. Preparation of Wound Fluid (WF)

WF was obtained from 6 patients after planned neck dissection at the Department of Otorhinolaryngology, Plastic, Aesthetic and Reconstructive Head and Neck Surgery at the Julius Maximilian University of Wuerzburg (Wuerzburg, Germany) after written informed consent (91/19-me). The procedure of extraction and processing of the fluid from vacuum drainages was carried out as previously described [32]. An exploratory preliminary experiment was conducted to identify the conditions regarding time interval and WF concentration, at which the strongest change in gene expression for pro-inflammatory genes was detectable after incubating MSC with WF in different concentrations and for different periods of time (for additional experimental details, see Supplementary Material). In this way, the combination of 30% WF and 3 h was determined. WF from 6 different patients was diluted in DMEM to a concentration of 30% for further experiments.

### 4.3. Measurement of CD4+ Cell Proliferation

1 × 10^5^ MSCs in 1 mL DMEM without supplements were applied to each well of a 12-well plate (Greiner Bio-One GmbH) and cultivated overnight. The following day, 2 × 10^5^ MSCs were washed in PBS and then incubated for 3 h with 30% WF at 37 °C and 5% CO_2_ and 2 × 10^5^ MSCs were incubated in DMEM expansion medium. Meanwhile, 2 × 10^6^ CD4+ PBMCs were suspended in 2 mL RPMI medium (Roswell Park Memorial Institute Medium, Biochrom AG, Berlin, Germany) with 1% penicillin/streptomycin (Sigma Aldrich), 1% HEPES (4-(2-Hydroxyethyl) piperazine-1-ethanesulfonic acid, Sigma Aldrich) and 10% FCS (Linaris). 10 µL IL-2-RPMI solution (Human IL-2 IS, Miltenyi Biotec) with a concentration of 50 IU IL-2 were added. After resuspension, the PBMCs were plated in 2 wells of a 12-well plate without MSCs. 2 µL PHA-L (phytohemagglutinin) (Biochrom AG), which served to stimulate the CD4+ lymphocytes, were added to each well. This 12-well plate served as a control plate.

13 × 10^6^ CD4+ PBMCs were first washed in RPMI medium and then in 1× PBS and subsequently centrifuged at 1400 rpm for 5 min. After removal of the supernatant, the cell pellet was resuspended in 26 mL PBS/5% FCS, resulting in a concentration of 5 × 10^5^ PBMCs/mL. 26 mL CFSE-PBS solution (5(6)-carboxyfluorescein diacetate N-succinimidyl ester (CFSE) (Sigma Aldrich)) with a concentration of 10 µM CFSE was prepared so that after mixing the cell suspension with the CFSE solution, the final CFSE concentration was 5 µM. The cells were incubated for 15 min in the dark at room temperature. The staining was stopped by adding the fivefold volume of PBS with 5% FCS, followed by another 5 min incubation in the dark at room temperature. Cells were now washed twice in PBS/5% FCS by centrifugation and then resuspended in RPMI medium. 6 × 10^6^ CFSE-stained CD4+ PBMCs were resuspended in 6 mL RPMI medium, and 30 µL IL-2. 2 × 10^6^ of these cells were applied on 2 wells of the control plate, and 2 µL PHA-L were then added to one of these wells. 8 μL of PHA-L was added to the remaining cell suspension.

After 3 h of WF incubation, MSCs were then incubated for 30 min with 1 mL of 25 µg/mL Mitomycin C solution (Mitomycin C from Streptomyces caespitosus) (Sigma Aldrich) to prevent further proliferation during co-cultivation. 4 × 10^6^ CFSE-stained CD4+ lymphocytes were divided into the 4 wells of the co-culture plate, with each well containing 1 × 10^5^ MSCs so that 1 × 10^6^ CD4+ cells were plated in 1 mL of RPMI medium per well. After 5 days of co-cultivation, FACS measurements were carried out. FACS tubes, which contained CFSE-stained CD4+ lymphocytes, were each mixed with 0.5 mL PBS and 0.5 µL Fixable Viability Dye 780 eFluor (Life Technologies Corp. (Applied Biosystems), Carlsbad, CA, USA) and incubated in the dark on ice for 30 min while the unstained cells were resuspended in 0.5 mL PBS. The cells of each FACS tube were resuspended in 300 µL PBS/1% FCS. Subsequently, the CFSE staining was measured by FACS. The cell culture supernatants were frozen at −80 °C. Flow cytometric analysis was performed using the FlowJo 10.2 software (BD Biosciences, Heidelberg, Germany).

### 4.4. Cytokine Measurements

#### 4.4.1. Quantitative Real-Time PCR of Cytokine Gene Expression in MSCs After WF Incubation

Gene expression was measured in MSC donors after incubation with 30% WF for 3 h. RNA extraction was carried out using the Qiagen RNeasy Mini Kit (Qiagen GmbH, Hilden, Germany) according to the manufacturer’s instructions. For cDNA synthesis, 4 µL of Master Mix (SuperScript^®^ VILO Mastermix, Life Technologies Corp. (Applied Biosystems), Carlsbad, CA, USA) were applied to Fast-Reaction-Tubes Micro Amp 8 Cap strips (Life Technologies Corp. (Applied Biosystems)). Then, the diluted RNA and DEPC-H_2_O were added to a total volume of 20 µL. The tubes were transferred to the RT-PCR device, StepOnePlus Real-Time PCR System (Life Technologies Corp. (Applied Biosystems). Once the cDNA had been prepared, it was mixed with the TaqMan^®^ Gene Expression Master Mix (Life Technologies Corp. (Applied Biosystems). The TaqMan^®^ Gene Expression Assays used: Interleukin-10 (IL 10/Assay-ID: Hs00961622_m1), Transforming growth factor-β1 (TGF-β1/Hs00234244_m1), Tumor necrosis factor-α (TNF-α/Hs02621508_s1) and Indoleamine 2,3-dioxygenase 1 (IDO-1/Hs00984148_m1). Glyceraldehyde-3-phosphate dehydrogenase (GAPDH/Hs02758991_g1) was used as the internal housekeeping gene. Results were quantified with the ΔΔCT method.

#### 4.4.2. Quantification of IDO-1 Concentration in Supernatants of Co-Culture by ELISA

The expression of IDO-1 was quantified using the human indoleamine 2,3-dioxygenase ELISA Kit (Novus Biologicals, Littleton, CO, USA). Supernatants of the co-culture of CD4+ lymphocytes and MSCs after 5 days were collected and ELISA was performed in accordance with the manufacturer’s instructions. The spectrophotometric evaluation was carried out on an ELISA microplate reader (BioTek Instruments GmbH, Bad Friedrichshall, Germany) at a wavelength of 450 nm.

#### 4.4.3. Measurement of TNF-α, TGF-β1, IFN-γ, and IL-10 in Supernatants of Co-Culture by Multi-Analyte Flow Assay Kit

Beads for TNF-α, TGF-β1, IFN-γ, and IL-10 were used for multiplex flow cytometry analysis using the Multi-Analyte Flow Assay Kit (LEGENDplex Human Essential Immune Response Panel Mix and Match Subpanel, Multi-Analyte Flow Assay Kit, Biolegend, San Diego, CA, USA). LegendplexTM Software (https://www.biolegend.com/Files/Images/BioLegend/legendplex/manuals/02-0022-02_software_Manual_r2.pdf (accessed on 26 December 2024)), a cloud-based online platform provided by the kit provider (Biolegend, San Diego, USA) was used to quantify the concentration of the substances in accordance with the fluorescence intensity.

### 4.5. Statistical Analysis

The obtained data were statistically analyzed using the GraphPad Prism software 5.01 (GraphPad Software, Inc., La Jolla, CA, USA). An ANOVA with a Bonferroni post hoc test and in case of no normal distribution, Kruskal–Wallis with Dunn’s post hoc test as a non-parametric method was implemented to calculate *p*-values, and *p*-values ≤ 0.05 were considered statistically significant.

## 5. Conclusions

This study demonstrates that MSCs shift the immunological environment toward anti-inflammatory conditions. Moreover, the findings indicate that WF-preconditioned MSCs exhibit an enhanced ability to suppress the proliferation of CD4+ cells compared to native MSCs. Notably, this research is the first to explore the impact of WF as a physiological stimulus on the immunomodulatory effects of MSC, offering preliminary data that could inform future investigations.

## Data Availability

The data that support the findings of this study are available from the corresponding author upon reasonable request.

## Supplementary Materials

The following supporting information can be downloaded at: https://www.mdpi.com/article/10.3390/ijms26010293/s1.
