# Supplementary material for: Preconditioning with Wound Fluid Enhances Immunosuppressive Properties of Mesenchymal Stromal Cells In Vitro
Source: Int J Mol Sci. 2024 Dec 31;26(1):293. doi: 10.3390/ijms26010293 (PMC11719632; doi:10.3390/ijms26010293)
Supplement: Supplementary file 1 [file ijms-26-00293-s001.zip › ijms-3367500-supplementary.pdf]

Gene expression was analyzed using RT-qPCR in three MSC donors, with each experiment performed in triplicate. MSCs were cultured in standard cell culture medium (DMEM with 1% Pen/Strep, without FCS), supplemented with either 30% or 60% WF for 3 hours or 24 hours. The expression levels of COX2, IL-6, and IL-8 were quantified at 0 hours, 3 hours, and 24 hours. As gene expression results showed minimal differences between the 30% and 60% WF concentrations at the 3-hour time point, the 30% concentration was selected for subsequent experiments to conserve WF, given its limited availability.
